# A systematic review and network meta-analysis on the optimal wavelength of low-level light therapy (LLLT) in treating knee osteoarthritis symptoms

**DOI:** 10.1007/s40520-024-02853-0

**Published:** 2024-10-05

**Authors:** Tianxiang Fan, Yang Li, Arnold Y. L. Wong, Xiao Liang, Yarou Yuan, Peng Xia, Zhi Yao, Deli Wang, Marco Y. C. Pang, Changhai Ding, Zhaohua Zhu, Ye Li, Siu Ngor Fu

**Affiliations:** 1 Department of Rehabilitation Sciences, The Hong Kong Polytechnic University, Kowloon, Hong Kong SAR China; 2 Clinical Research Centre, Zhujiang Hospital, Southern Medical University, Guangzhou, Guangdong China; 3 Research Institute for Smart Ageing, The Hong Kong Polytechnic University, Kowloon, Hong Kong SAR China; 4 Department of Rehabilitation Medicine, Nanjing First Hospital, Nanjing Medical University, Nanjing, Jiangsu China; 5 Department of Bone and Joint Surgery, Peking University Shenzhen Hospital, Shenzhen, Guangdong China

**Keywords:** Low-level light therapy, Osteoarthritis, Knee pain, Meta-analysis

## Abstract

**Objectives:**

To compare the efficacy of the various wavelengths of low-level light therapy (LLLT) in alleviating knee pain, dysfunction, and stiffness in patients with knee osteoarthritis (KOA), and to compare the effectiveness of LLLT versus sham treatment in reducing knee pain, dysfunction, and stiffness.

**Methods:**

PubMed, Web of Science, EMBASE, and Cochrane Library were searched from inception to 12 December 2023. Randomized controlled trials that assessed the effects of different wavelengths of LLLT on alleviating pain of patients with KOA were included. A conventional meta-analysis and network meta-analysis were preformed, and standardized mean differences (SMD) with 95% confidence interval (CI) were calculated.

**Results:**

Thirteen studies involving 673 participants with KOA met inclusion criteria. Overall, LLLT was superior to sham LLLT for relieving pain (SMD = 0.96, 95% CI 0.31–1.61) but not for improving function (SMD = 0.21, 95% CI − 0.11 to 0.53) or stiffness (SMD = 0.07, 95% CI − 0.25 to 0.39). Surface under the cumulative ranking curve (SUCRA) value ranking showed the most effective wavelength of LLLT in reducing KOA pain was 904–905 nm (SUCRA, 86.90%), followed by multi-wavelengths (MWL) (SUCRA, 56.43%) and 785–850 nm (SUCRA, 54.97%). Compared to sham LLLT, L2 (SMD = 1.42, 95% CI = 0.31–2.53) and L1 (SMD = 0.82; 95% CI = 0.11–1.50) showed a significant reduction in KOA pain. However, MWL (SMD = 0.83; 95% CI = − 0.06 to 1.72) showed similar KOA pain reduction compared to sham LLLT. The certainty of evidence showed that the quality of evidence regarding the effectiveness of overall LLLT versus sham, and 904–905 nm versus sham were low, while the quality of evidence for MWL versus sham, and 785–850 nm versus sham was very low.

**Conclusion:**

While the 904–905 nm wavelength showed potential benefits in reducing KOA pain, the overall quality of the evidence was low. LLLT with 904–905 nm or 785–850 nm wavelengths yielded significantly better reduction in KOA pain compared to sham LLLT, but further high-quality research is warranted to validate these findings.

**Supplementary Information:**

The online version contains supplementary material available at 10.1007/s40520-024-02853-0.

## Introduction

Knee osteoarthritis (KOA) is a highly prevalent chronic joint disease affecting over 654 million people worldwide, incurring enormous societal costs, particularly among older adults[1, 2]. KOA commonly manifests as joint pain, stiffness, and functional limitations, leading to reduced mobility and quality of life [3–5]. With an aging and increasingly obese population, the prevalence of KOA is expected to rise [3].

Low-level light therapy (LLLT) is considered a potential non-pharmaceutical therapy for knee OA. LLLT uses laser and/or light-emitting diode (LED) with specific wavelengths of light to stimulate cellular processes, modulate inflammation, and promote tissue healing [6–9]. Given the non-invasiveness, rapid pain relief, and minimal side effects of LLLT, it is widely used to treat various musculoskeletal disorders [6]. LLLT has shown promise as a therapeutic intervention for OA by modulating inflammation, suppressing the expression of pain-associated molecules, and improving pain behavior in animal models of OA [10–13]. Conflicting findings have been reported in clinical trials regarding the effectiveness of LLLT in treating OA [14–16]. Some trials demonstrated that LLLT was significantly better than sham LLLT in relieving pain [17–19], while others found no significant pain reduction [20, 21]. Similarly, systematic reviews also revealed mixed results regarding the efficacy of LLLT plus exercise therapy in alleviating pain in individuals with Knee OA (KOA) [14, 22, 23]. There is also no consensus on whether standalone LLLT can improve OA-related pain [14, 24]. These inconsistencies may be attributed to variations in treatment parameters, such as wavelengths, dosages, and duration of LLLT, as well as methodological limitations of previous studies. Although a systematic review and meta-analysis conducted in 2015 reported no significant superiority of LLLT over sham LLLT in improving visual analogue scale (VAS) pain, Western Ontario and McMaster Universities Arthritis Index scores (WOMAC) pain, WOMAC stiffness, or WOMAC function, this review had several limitations [14]. It analyzed VAS pain scales and WOMAC pain scale separately, reducing the sample size and increasing the risk of false-negative results, and excluded some KOA studies using alternative pain scales. Given these limitations and the publication of seven new clinical trials since 2015, there is a clear need for an updated systematic review and meta-analysis to comprehensively summarize the current evidence in this field.

The effectiveness of LLLT depends on multiple factors, including wavelengths of light, energy density, and/or total energy. Given the diverse settings of various treatment parameters in prior studies, they might have confounded the findings and interpretation of the effectiveness of LLLT [25]. It is noteworthy that the wavelength of light not only affects the penetration depths of tissues but also plays a critical role in eliciting different biological effects [6, 26]. Some randomized control trials (RCTs) and a meta-analysis have shown that different wavelengths of LLLT lead to differential clinical outcomes. Specifically, Jankaew et al. found that the 808 nm wavelength group showed significantly better results in knee extensor strength compared to the 660 nm group in patients with KOA [27]. Additionally, Ahmad et al. reported that the 1064 nm LLLT combined with exercise led to significantly better improvements in pain, physical function, and knee-related disability than the 830 nm LLLT combined with exercise. Furthermore, a meta-analysis found that wavelengths of 785–860 nm or 904 nm significantly alleviated pain and disability in patients with KOA [28]. However, no network meta-analysis has compared the relative effects of different wavelengths of LLLT on pain, stiffness, and function in patients with KOA. Therefore, a better understanding of the specific wavelength range for yielding optimal clinical outcomes is crucial for effective LLLT treatments. The World Association for Photobiomodulation Therapy (WALT) recommends using LLLT wavelength range of 780–860 nm and 904 nm for treating musculoskeletal disorders [29]. However, the relative efficacy of these two wavelength ranges in treating OA symptoms is unclear. Further, compared to LLLT of a single radiation, a combination of LLLT of different wavelengths may generate distinct effects on different biological tissues. However, no prior meta-analysis has compared the relative efficacy of multi-wavelengths (MWL) of LLLT, and various separate wavelengths of LLLT in reducing OA symptoms. This research gap can be addressed by evaluating the efficacy of MWL LLLT and single-wavelength LLLT through a network meta-analysis that compares multiple interventions using direct and indirect comparisons to help rank the comparative effectiveness of various treatments [30].

Given the above, the primary objective of the current systematic review and meta-analysis was to summarize the efficacy of LLLT compared to sham LLLT in improving knee pain, stiffness and function in patients with KOA. The secondary objective was to conduct a network meta-analysis to compare the efficacy of different wavelengths and MWL in reducing pain, stiffness, and improving function in patients with KOA, to identify the most effective wavelength range.

## Methods

### Protocol and registration

This systematic review and network meta-analysis was registered with PROSPERO (ID: CRD42023396103). The reporting of this review followed the Preferred Reporting Items for Systematic Reviews and Meta-Analyses guidelines.

### Search strategies and selection criteria

Four electronic databases (PubMed, Web of Science, EMBASE, and Cochrane Library) were searched from inception to 12 December 2023. The detailed search strategies are shown in Supplementary Table 1.

The following inclusion criteria were adopted based on the PICOS framework: (1) Study design: Randomized control trials (RCTs); (2) Patients: Patients with knee OA on either side; (3) Intervention: LLLT was found in at least one of the treatment groups; (4) Comparator: The intervention in the control group might use sham LLLT or another wavelength of LLLT; and (5) Outcomes: Pain intensity as evaluated by the WOMAC, VAS, Numeric Pain Rating Scale (NPRS), or Visual Numerical Scale (VNS) was set as primary outcome. WOMAC knee function and stiffness scores were set as the secondary outcomes.

Exclusion criteria: (1) missing LLLT wavelength; (2) non-English language articles; (3) LLLT not using laser or LED; (4) Review articles or meta-analyses; (5) studies without a control group; (6) animal or cell studies; (7) unrelated to KOA; (8) the involvement of other therapies adjunctive to LLLT; (9) no reporting of knee pain, knee physical function or knee stiffness; (10) abstracts, conference proceedings, grey literature or studies without extractable data.

Identified citations were imported to EndNote 20. Two independent reviewers (TF and YY) screened potential titles and abstracts based on the selection criteria. Any disagreements were discussed with a third reviewer (YL). Eligible abstracts were retrieved for full-text screening. The same procedure was applied for the full-text screening. Relevant full texts were included. Excellent inter-reviewer reliability of the screening was noted (kappa coefficient = 0.95). The reference lists of the included studies were screened for relevant articles. Forward citation tracking was conducted using the Web of Science. The corresponding authors of the included studies were contacted by emails to seek pertinent articles.

### Data extraction

Two independent reviewers (TF and YY) extracted data The extracted data included authors, year of publication, study design, sample size, age, body mass index, and gender. Additionally, details of LLLT treatment such as wavelengths, total energy, energy density, follow-up time, and treatment frequency were recorded. Clinical outcomes comprised the baseline mean, baseline standard deviation, follow-up mean, and follow-up standard deviation for pain, physical function, or stiffness. If standard deviations were not reported, we extracted 95% confidence intervals (CIs), standard errors (SEMs), or data from error bar graphs. When only graphical data were available, numerical data were extracted using the Engauge Digitizer 12.1 software. Any disagreements between reviewers were resolved through discussion and consensus; if needed, a third independent reviewer (YL) made the final decision. To ensure data accuracy and completeness, quality checks were conducted on a random subset of the extracted data by an independent reviewer (YL). Based on the intervention wavelengths recommended by WALT, LLLT wavelengths were divided into two categories: 785–860 nm (L1) and 904 nm (L2). Since a study used the wavelength of 905 nm, which is very close to 904 nm, the findings were considered as the same group (904 nm-905 nm, L2). The treatment group that used more than one wavelength of light was categorized as the multi-wavelength (MWL) group.

Pain intensity evaluated by WOMAC and/or visual pain scale (VAS, NPRS, or VNS), WOMAC knee function and stiffness scores were the potential clinical outcomes. In the assessment of pain within the same study, when various pain scales were used, we prioritized the analysis of pain scale using the following sequence: the WOMAC pain scale, the VAS pain scale, the NPRS pain scale, and finally, the VNS pain scale [31].

Reviewers calculated the effect sizes by measuring the mean difference (MD) and standard division (SD). A study compared the effects of LLLT and sham LLLT on pain at different time points. The findings revealed a significant difference in pain pressure threshold at the 8th week, suggesting that 8 weeks may be suitable time for LLLT intervention [32]. For pain, physical function, and stiffness, the time point was at or nearest to 8 weeks after initial LLLT or sham LLLT [33].

Specifically, we extracted the original MDs and SDs in each included study, if available. When only standard errors (SEs) were reported, we converted them to SDs according to the Cochrane Handbook for Systematic Reviews [34]. For graphical information, numerical data was extracted using the Engauge Digitizer 12.1 software (Mark Mitchell, Palos Verdes Peninsula, CA, USA) [35]. When only the baseline and follow-up data rather than the MDs and SDs were available, we calculate the MDs by subtracting the follow-up mean from the baseline mean. For studies that did not provide the SDs of the outcome changes, the SDs were estimated using following equation with a correlation coefficient (r) of 0.5:

$${SD}_{change = \sqrt{{{SD}_{baseline}^{2}}+ {SD}_{follow-up}^{2} - (2r \times {SD}_{baseline} \times { SD}_{follow-up}}}$$

according to the Cochrane Handbook guideline [34]. If none of these options were feasible, the corresponding authors of the included study were contacted by emails for a maximum of three times.

### Quality assessments

Two independent reviewers (TF and YL) used the Cochrane risk-of-bias tool for randomized trials (RoB 2) to evaluate the methodological quality of the included studies by [36]. The tool evaluates six domains: (1) randomization process; (2) deviation from intended interventions; (3) missing outcome data; (4) measurements of outcomes; (5) selection of the reported results; and (6) overall bias. Each domain was classified as low, some concerns, or high risk of bias based on criteria from the Cochrane Handbook for Systematic Reviews [36]. Discrepancies between reviewers were resolved through discussion and consensus. If consensus could not be reached, the final decision was made after discussion with the third reviewer (YY). The overall bias of each study was corresponds to the worst risk of in any of the first five domains, as suggested by the BMJ guideline [36].

### Certainty of the evidence

The grading of recommendation assessment, development, and evaluation (GRADE) approach was utilized in this meta-analysis to assess the certainty of evidence [37]. Two independent reviewers (TF and PX) conducted assessments for each comparison and resolved any discrepancies through consensus. Certainty ratings were assigned for each comparison and endpoint, with ratings of high, moderate, low, or very low, based on a thorough evaluation of risk of bias, inconsistency, indirectness, publication bias, intransitivity, incoherence and imprecision according to the GRADE handbook [38].

## Statistical analyses

All the calculations and figures drawing were conducted in R software, version 4.1.3. Conventional meta-analyses comparing LLLT with sham LLLT were conducted for each KOA symptomatic outcomes using “meta” and “metafor” packages [39, 40]. The “meta” package’s “metacont” function was used to calculate common and random effects estimates for meta-analyses with continuous outcome data, utilizing inverse variance weighting for pooling. The ‘forest’ function from the “metafor” package generated forest plots based on results calculated by “metacont”. A random effects model network meta-analyses were then conducted to explore the relative efficacy of LLLT with different wavelengths, using the “netmeta” package based on the frequentist framework [41]. The consistency between direct and indirect comparisons was tested by node-splitting analysis.

The *I*^2^ test was conducted to assess the heterogeneity of each pairwise comparisons by “metacont”, with *I*^2^ exceeding 50% indicating heterogeneity[42]. Because the included studies used slightly different scales for measuring various clinical outcomes, changes from baseline to follow-up were converted to standardized mean difference (SMD). SMDs were calculated as the difference in mean outcomes between groups divided by the standard deviation of the outcome among participants [34]. Regarding the calculation of standard deviations, we employed the pooled standard deviation method. We first calculated the baseline and follow-up standard deviations for both the experimental and control groups. Then, the standard deviation change in each group was incorporated into the “metacont” function in R for further calculation using a pooled standard deviation.

To numerically rank the associations between all interventions and pain reduction, the surface under the cumulative ranking curve (SUCRA) was calculated [43]. The value of SUCRA ranges from 0–100%. The SUCRA values represent the probability of an intervention being ranked as the best, second best, and so on, for the outcome of interest. A SUCRA value of 100% indicates that the intervention is certain to be the most effective, while a value of 0% indicates that it is certain to be the least effective.

To assess publication bias, the Egger regression test and the funnel plot were employed using the “meta” package. In this analysis, a *p*-value less than 0.10 was considered significant, indicating the presence of asymmetry and publication bias [44]. Sensitivity analysis was conducted by excluding studies with high risk of overall bias. Two-sided tests were conducted for all analyses, and statistical significance was defined as a *p*-value less than 0.05.

## Results

### Study selection

**Fig. 1:**
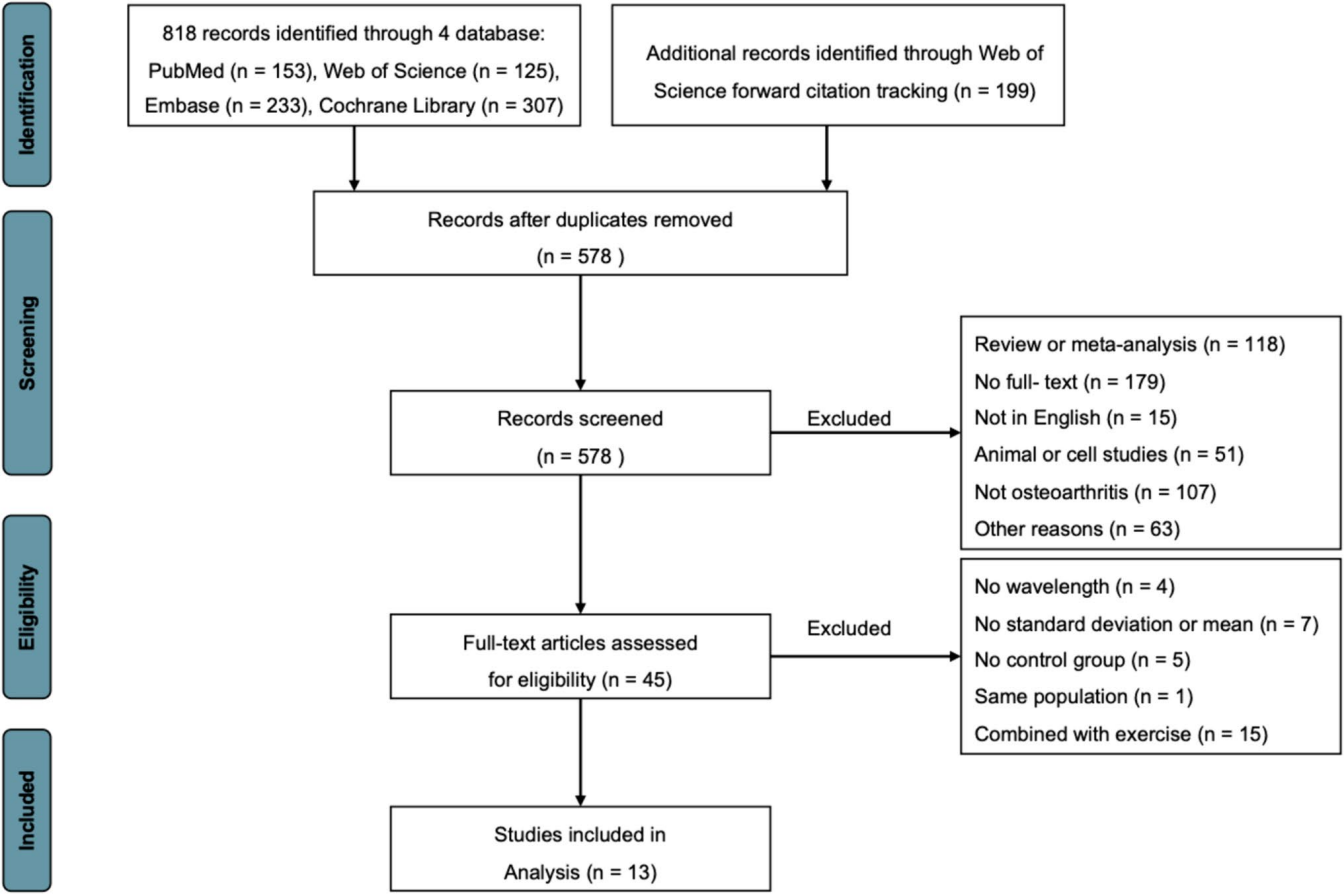
A flow diagram of the study identification and selection process

A total of 818 records were identified in the initial search from four databases and 199 records from the forward citation tracking in Web of Science. Figure 1 illustrates the screening procedure and the number of articles that met the inclusion criteria. After removing 439 duplicates, 578 articles were screened for titles and abstracts. Finally, a total of 13 RCTs were included in the analysis (Fig. 1) [17, 19–21, 45–53]. A total of 12 studies employed lasers as the light source for LLLT, while one study conducted by Nathali Cordeiro Pinto et al. utilized LED light source [47].

### Study characteristics

Table 1 shows the demographic characteristics of the included studies. A total of 673 participants from 13 trials were included in the analysis, the sample size of these trials ranged from 29 to 101. The mean age of included patients ranged from 55.2 to 69.0 years. All participants were categorized into four groups according to their received treatments: 785–850 nm LLLT (L1), 904–905 nm LLLT (L2), multi-wavelength LLLT group (MWL), sham LLLT (sham). All included studies evaluated knee pain. Four included studies assessed knee physical function and stiffness. Eight included studies were double-blinded RCTs, while five trials were single-blinded RCTs.

**Table 1:** Characteristics of the included studies

First author, Publication year	Study Design	Sample size	Age (years)	Female (%)	BMI	Follow-up (week)	Frequency, duration/session, number of sessions	Wavelength, type of device	Treatment area	Total energy/session	Energy density (J/cm^2^)	Primary outcome	Secondary outcome
Punpetch Siriratna, 2022 [48]	SB-RCT	21	66.1 ± 9.4	85.7	28.1 ± 5.2	4	2–3 sessions/week for a total, 8 min/session, 10 sessions	808 nm + 905 nm, laser	8 points around the knee joint	562.50	22.39	VAS pain	–
		21	65.0 ± 8.5	76.2	27.4 ± 5.8			0		0	0		–
Renata Alqualo-Costa, 2021 [49]	DB-RCT	42	61.3 ± 9.4	83%	31.2 ± 6.1	4	3 sessions/week, 75 s/session, 12 sessions	904 nm, laser	Pain areas	27	54	VAS pain	–
		42	65.3 ± 8.5	78%	29.9 ± 4.6			0		0	0		
Patricia Gabrielli Vassão, 2020 [50]	DB-RCT	14	64.00 ± 4.93	–	29.08 ± 4.81	8	2 sessions/week, 40 s/session, 16 sessions	808 nm, laser	7 points around knee	56	91	NPRS pain	–
		15	65.37 ± 4.19	–	27.52 ± 3.31			0		0	0		
Roberta de Matos Brunelli Braghin, 2019 [20]	DB-RCT	15	58.20 ± 7.97	87%	31.57 ± 3.58	8	2 sessions/week, 56 s/session, 15 sessions	808 nm, laser	10 points around knee	200	56	WOMAC pain	WOMAC stiffness, WOMAC physical function
		15	60.8 ± 9.2	80%	26.52 ± 4.43			0		0	0		
Gopal Nambi S, 2017 [14]	DB-RCT	17	58 ± 6	–	26.9 ± 4.8	4	3 sessions/week, 60 s/session, 12 sessions	905 nm, laser	8 points around the knee	12	1.5	VAS pain	–
		17	60 ± 8	–	28.3 ± 3.5			0		0	0		
Ahmad Alghadir, 2014 [18]	SB-RCT	20	55.2 ± 8.14	50%	32.34 ± 5.77	4	2 sessions/week, 10 min/session, 8 sessions	850 nm, laser	8 points around the joint	48	48	WOMAC pain	WOMAC stiffness, WOMAC physical function
		20	57 ± 7.77	40%	33.09 ± 4.98			0		0	0		
Nelson Marquina, 2012 [19]	SB-RCT	53	-	41.50%	–	6	3 sessions/week, 1 min/session, 12 sessions	905 nm + 660, laser nm	7 points around the joint	1.68	24	VAS pain	–
		48	-	31.25%	–			0		0	0		
Kamila Gworys, 2012 [52]	DB-RCT	31	67.7 ± 11.3	–	–	2	5 sessions/week,-, 10 sessions	0		0	0	VAS pain	–
		34	57.6 ± 11.8	–	–			810 nm, laser	7 points around the joint	152.4	12.7		
		31	65.4 ± 9.6	–	–			808 nm + 905, laser nm		148.8	6.21		
Funda Tascioglu, 2004 [21]	SB-RCT	20	62.86 ± 7.32	70%	27.56 ± 5.65	3	5 sessions/week, 10 min/session, 10 sessions	830 nm, laser	5 points around the joint	15	382	WOMAC pain	WOMAC stiffness, WOMAC physical function
		20	64.27 ± 10.55	65%	29.56 ± 9.54			0		0	0		
Vanessa Ovanessian Fukuda, 2011 [53]	DB-RCT	25	63.0 ± 9.0	20%	30.0 ± 3.5	3	3 sessions/week, 7.5 min/session, 9 sessions	904 nm, laser	9 points around the joint	27	6	VNPS pain	-
		22	63.0 ± 8.0	36%	28.7 ± 4.1			0		0	0		
Xueyong Shen, 2009 [45]	SB-RCT	20	60.10 ± 6.83	10%	–	4	3 sessions/week, 20 min/session, 12 session	650 nm + 1060 nm, laser	Acupuncture point Dubi (ST 35)	-	-	WOMAC pain	WOMAC stiffness, WOMAC physical function
		20	56.40 ± 7.41	10%	–			0		0	0		
Dwi R. Helianthi, 2016 [46]	DB-RCT	30	69 ± 6.0	60%	25.8 ± 4.3	6.5	2 sessions/week, 5 min 20 s/session, 10 sessions	785 nm, laser	Acupuncture points of ST35 Dubi, ST36 Zusanli, SP9 Yinlingquan, GB34 Yanglingquan and EX-LE-4 Neixiyan	20	10	VAS pain	–
		29	68 ± 5.0	60%	26.3 ± 4.3			0		0	0		
Nathali Cordeiro Pinto, 2022 [47]	DB-RCT	15	63 ± 10.9	93%	24.8 ± 9.6	8	2 sessions/week, 5–8 min/session, 10 sessions	850 nm, light-emitting diode	Whole joint surface	526–1402	18–48	NPRS pain	–
		16	66 ± 10.7	94%	29.8 ± 4.6			0		0			

*DB-RCT* Double blind-randomized controlled trial, *SB-RCT* Single blind-randomized controlled trial, *BMI* Body mass index, *WOMAC* Western Ontario and McMaster Universities Arthritis Index scores, *VAS* Visual analogue scale, *NPRS* Numeric pain rating Scale, *VNS* Visual numerical scale

Mean (standard deviation) are provided above for age, BMI

### Risk of *bias* assessments

**Fig. 2:**
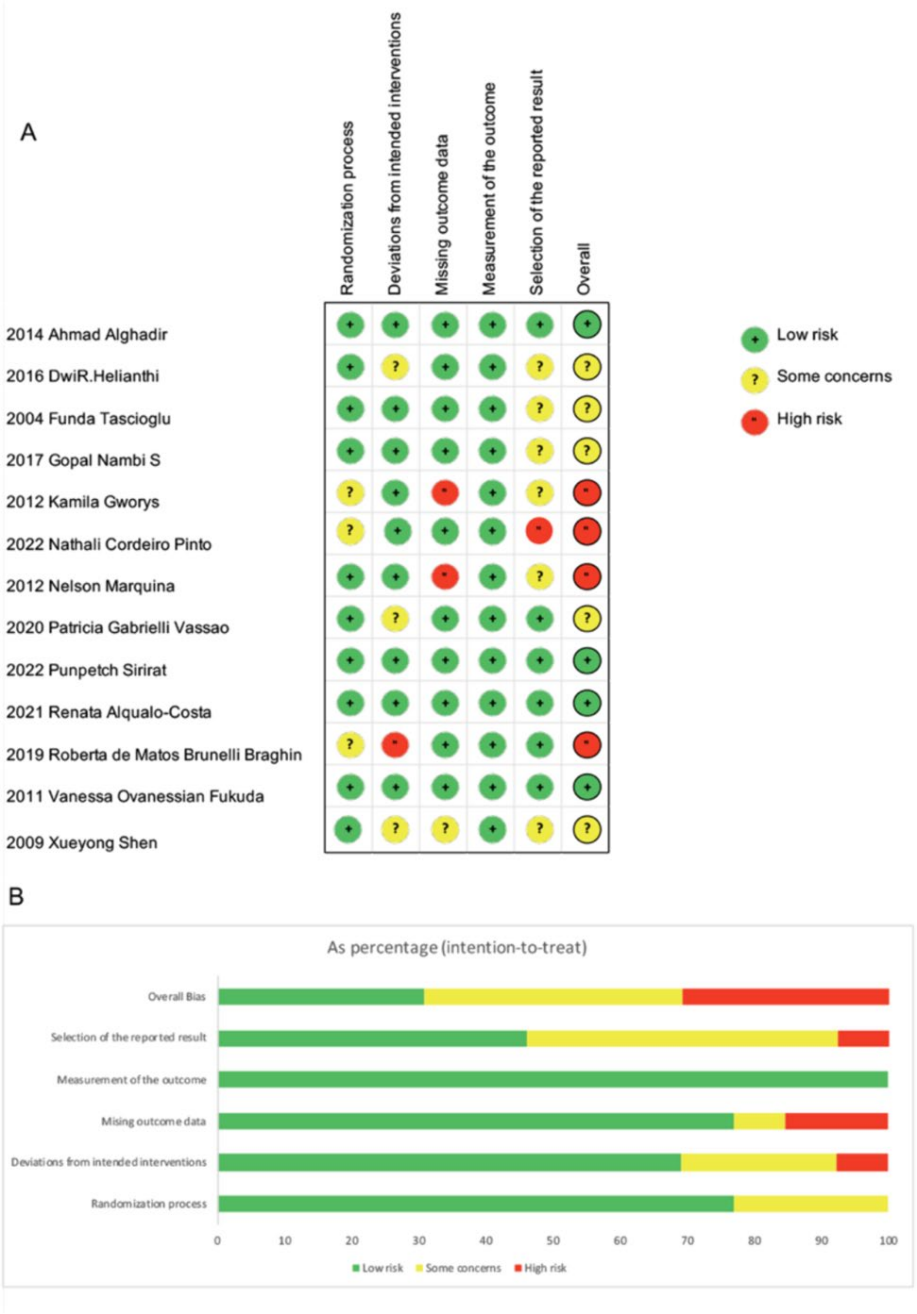
Risk of bias assessments. **A** the judgement of each bias item for each included study. **B** judgements of each bias item presented as percentages across all included studies

Figure 2 depicts the risk of bias assessments of the included studies. Two studies had a high rate of missing values, which skewed the results due to inadequate analysis methods. Another study was deemed high risk because participants may have been aware of their assigned intervention during the trial. Additionally, five studies were rated as having some concerns. These studies had some concerns with deviations from intended interventions, selective reporting of results, or some missing outcome data. Although these concerns were not severe enough to classify the studies as high risk, they indicated minor inconsistencies in intervention administration, selective outcome reporting, and inadequate handling of missing data. Four studies demonstrated a low risk of bias. The risk-of-bias summary and graph are shown in Fig. 2.

### Conventional *meta*-analyses of the effects of LLLT interventions on knee pain, physical function, and stiffness

**Fig. 3:**
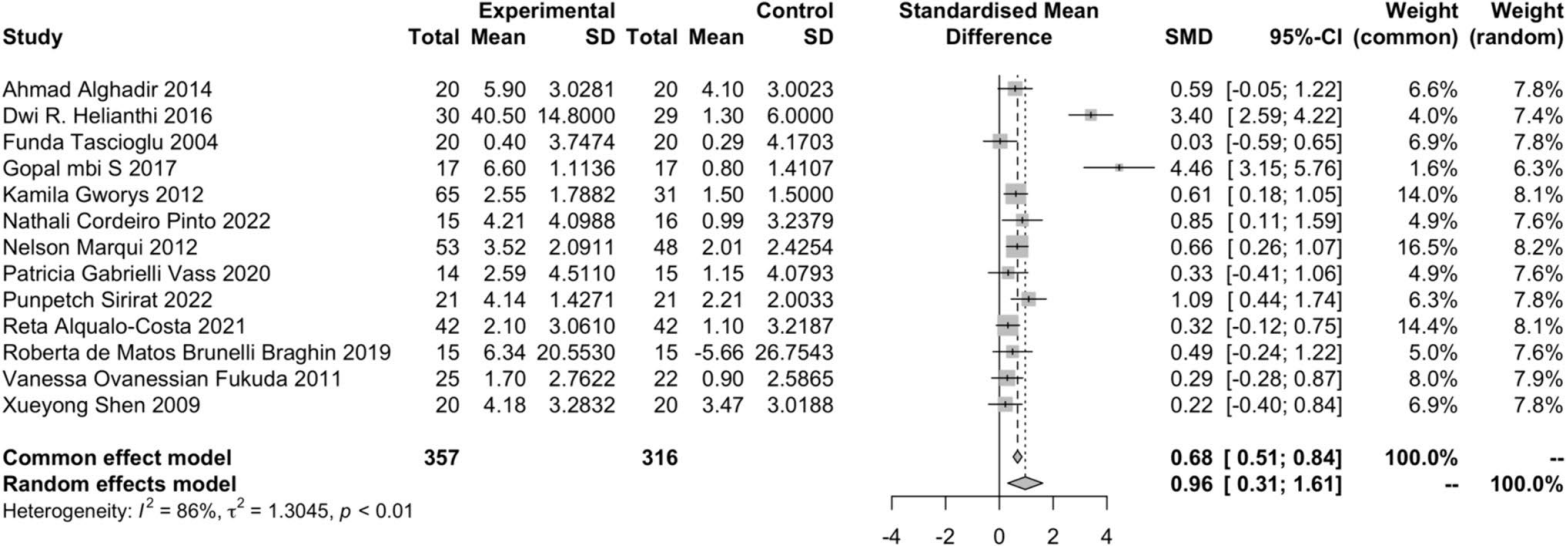
A forest plot of evidence from the direct comparisons between LLLT and sham LLLT for knee pain. *SMD* standardized mean difference, *SD* standard division

Findings from 13 included studies showed that LLLT was superior to sham LLLT for pain relief (SMD = 0.96, 95% CI 0.31–1.61) (Fig. 3). There is low certainty of evidence supporting overall LLLT for knee pain, primarily due to one trial showing a high risk of bias. Additionally, significant heterogeneity across 13 studies was found in meta-analysis, as indicated by an I^2^ value of 86%. Similar concerns apply to the effectiveness of 904–905 nm LLLT, with an I^2^ of 95%. Furthermore, findings from network meta-analysis also decreased the certainty of evidence. Evidence for the effectiveness of multiwavelength LLLT was very low, as indicated by the 95% CI of SMD including zero, alongside results from network meta-analysis. Additionally, there is very low certainty in the effectiveness of 785–850 nm LLLT for knee pain, compounded by missing data in one trial, an I^2^ of 88.8%, and evidence from network meta-analysis. (Supplementary Table 2 & Supplementary Fig. 1). Additionally, the results from four included studies showed that LLLT was not significantly better than sham LLLT for knee function (SMD = 0.21, 95% CI − 0.11 to 0.53) or knee stiffness (SMD = 0.07, 95% CI − 0.25 to 0.39) (Supplementary Figs. 6 and 7). The results of the sensitivity analysis that excluded high-risk studies for knee pain consistently showed that LLLT was superior to sham LLLT for pain relief (SMD = 1.13, 95% CI 0.15–2.12) (Supplementary Fig. 4).

### Network *meta*-analysis of the effects of LLLT interventions on knee pain, physical function, and stiffness

**Fig. 4:**
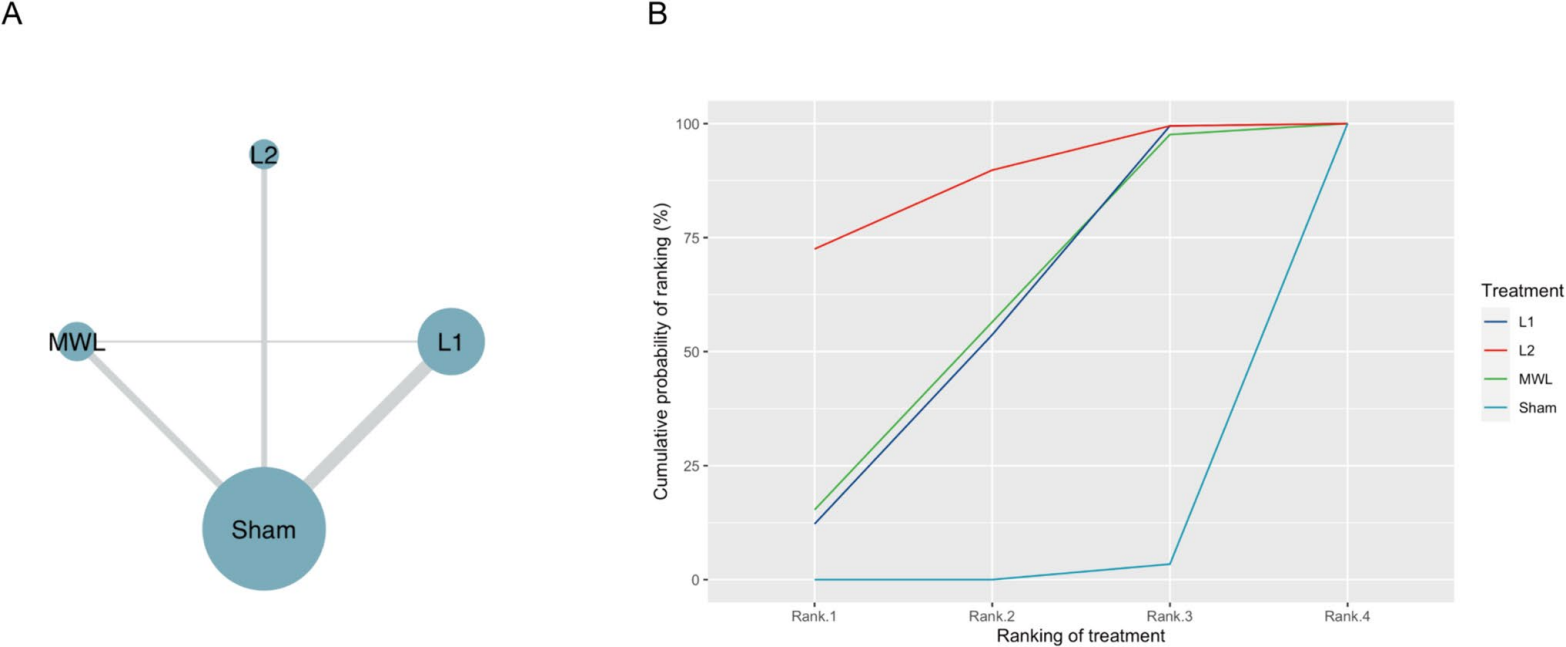
A network plot and Surface under the cumulative ranking curve (SUCRA) plot for knee pain. **A** Different nodes represent different intervention groups. The size of the nodes is proportional to the number of patients who were assigned to the intervention. The thickness of the lines connecting the nodes is proportional to the number of pairwise trials that evaluated the interventions. **B** SUCRA plot. L2 has the largest probability to be the first best treatment for knee pain. *L1* 785–850 nm LLLT, *L2* 904–905 nm LLLT, *MWL* multi-wavelength LLLT, *Sham* sham LLLT, *LLLT* low-level light therapy

Thirteen studies were included in the network meta-analysis to compare the relative effectiveness of different wavelengths of LLLT and sham LLLT in improving knee pain. Compared with sham LLLT, L2 (SMD = 1.42, 95% CI = 0.31–2.53) and L1 (SMD = 0.82; 95% CI = 0.11–1.50) significantly improved knee pain, while MWL (SMD = 0.83; 95% CI = − 0.06 to 1.72) did not show a significant effect (Table 2). However, L2 was not superior to L1 or MWL in relieving knee pain. The rank plot recommends L2 (SUCRA, 86.70%) seems to be the most effective therapy for knee pain relief, followed by MWL (SUCRA, 57.01%) and L1 (SUCRA, 54.97%) (Fig. 4). The results of the sensitivity analysis after excluding high-risk studies showed that only L2 (SMD = 1.56, 95% CI = 0.02–3.09) was effective in relieving knee pain (Supplementary Fig. 5). Node-splitting analysis indicated a good global and local consistency of the network for knee pain (Supplementary Table 2).

**Table 2:** Network meta-analysis of different wavelengths of LLLT with Surface under the cumulative ranking curve (SUCRA) value for knee pain

			**SUCRA value**
**L1**			86.90%
− 0.61 (− 1.92, 0.70);	**L2**		54.97%
− 0.02 (− 1.08, 1.04)	0.59 (− 0.83, 2.01)	**MWL**	56.43%
**0.81 (0.11, 1.50)**	**1.42 (0.31, 2.53)**	0.83 (− 0.06, 1.72)	**Sham LLLT**

League tables showing the results of the network meta-analyses, with the different standardized mean difference (SMD) and 95% credible intervals in the lower left part of the table, and the SUCRA values presented in the upper right part

Bold numbers are statistically significant

*LLLT* low-level light therapy, *L1* 785–850 nm LLLT, *L2* 904–905 nm LLLT, *MWL* multi-wavelength LLLT, *Sham* sham LLLT

For the included studies that assessed knee physical function and stiffness, only L1, MWL or sham LLLT were used as interventions. Neither L1 nor MWL showed significant improvement in physical function or knee stiffness compared to sham LLLT (Supplementary Figs. 6 & 7).

## Discussion

This systematic review and network meta-analysis is an update of a previous systematic review, and found low-certainty evidence to support LLLT was effective for alleviating KOA pain at eight weeks post-treatment. Specifically, LLLT with both L1 and L2 wavelength was superior to sham LLLT in knee pain reduction. According to SUCRA ranking, L2 was the optimal wavelength of LLLT in reducing knee pain, followed by MWL and L1. LLLT of any wavelength was found no effect on KOA function and stiffness.

Several previous meta-analyses revealed inconsistent results regarding the effects of LLLT on KOA. A meta-analysis by Huang et al. found no significant effect of LLLT on knee pain, function, or stiffness [23]. Their search was out of dated (conducted between January 2000 and November 2014), resulting in only nine included studies with relatively small sample sizes [23]. Conversely, a meta-analysis including 14 studies found that LLLT significantly improved pain, function, and stiffness in individuals with KOA [23]. However, this meta-analysis did not identify the detailed parameters of LLLT (e.g., wavelengths), leading to significant heterogeneity in the result [23]. Different wavelengths of LLLT not only exhibit varying tissue penetration capabilities, but also induce different biological effects [54, 55]. The inconsistent findings across different studies investigating LLLT for treating KOA might be attributed to the variations in LLLT parameters [56]. A Cochrane Library review highlighted the importance of consistent reporting of the characteristics of LLLT devices [56]. Despite the positive findings of some studies, previous meta-analyses lacked data on how the effectiveness of LLLT is influenced by various important factors, including wavelength, duration, dosage, and treatment site [56]. Prior to this review, the optimal treatment parameters for KOA using LLLT were unclear. Although the WALT gave recommendations on the wavelength for phototherapy, there was insufficient evidence to support their suggested wavelength for OA [29]. Our analysis revealed a significant reduction in KOA pain using LLLT with wavelength of 785–850 nm or 904–905 nm. According to SUCRA ranking, 904–905 was the optimal wavelength of LLLT in reducing knee pain, followed by MWL and L1. However, it is important to note that the confidence intervals for the SMDs overlap considerably, indicating a degree of uncertainty in the comparative effectiveness of these wavelengths. Further research is needed to confirm these findings and to establish the optimal wavelengths for LLLT in the treatment of KOA. Additionally, we did not observe significant pain reduction with MWL. Our findings provide low-certainty evidence to support the selection of optimal wavelength for future LLLT treatment of KOA. A prior systematic review found that LLLT was beneficial as an adjunct treatment to exercise in Incoherence managing KOA[57]. However, our review excluded studies containing treatment groups that combined exercise therapy with LLLT due to heterogeneous exercise protocols. The heterogeneity in exercise protocols could substantially impact the model's transitivity in a network meta-analysis, leading to unreliable results in indirect comparisons [34]. Transitivity is a crucial assumption, indicating that included studies should be similar in their distributions of effect modifiers. Violating this assumption can result in misleading indirect comparisons. Differences in exercise protocols across studies can introduce variability that affects the comparability of interventions, potentially biasing estimates of treatment effects and undermining the reliability of conclusions. The observed superior effectiveness of L2 compared to L1 in reducing pain may be attributed to the potential mechanism involving the stronger penetration of LLLT with wavelengths 904–905 nm compared to 785–850 nm [58]. The 904-905 nm wavelength of LLLT, known for its deeper tissue penetration, may benefit patients with severe cartilage damage and obesity-related KOA. Its enhanced penetration is crucial for treating deep joint cartilage damage effectively, making it particularly advantageous for obese patients [59].

Although LLLT with wavelengths 785–850 nm may reduce inflammation and pain [60, 61], LLLT at such wavelengths has difficulty in penetrating the knee joint. This may lead to limited biological responses due to absorption by the joint tissues, affecting the efficacy. Furthermore, multi-wavelength LLLT did not show significant difference in pain reduction as compared to sham LLLT. One possible reason is that different studies used different combinations of wavelengths as their multi-wavelength LLLT, which might confound the findings. Another possible reason could be that some studies in the multi-wavelength LLLT group used very low energy or energy density [19, 52], which may not be sufficient to induce significant biological effects. Interestingly, despite the larger SMD favoring L2 over L1, there was no significant difference between these two groups. This may be attributed to the fact that the comparisons between L2 and L1 were based on indirect evidence, resulting in a high degree of incoherence and imprecision in the results. Regarding LLLT on stiffness and physical function, both the conventional meta-analysis and network meta-analysis did not find a significant effect compared to sham LLLT. This could be due to the absence of studies specifically investigating stiffness and physical function using the 904–905 nm wavelength. Further clinical trials are warranted to explore these aspects in the future. The inclusion of a multi-arm study included into our network meta-analysis, which could impact results in several ways. Firstly, a multi-arm study provides direct comparative data between two treatment intensities, increasing the number of direct comparisons within the analysis and potentially enhancing the statistical power and certainty of evidence for those treatments [62]. Secondly, a multi-arm study enhances network connectivity by linking treatments that might only be indirectly connected through other paths, which could improve the accuracy and stability of overall network estimates [63]. Finally, the likelihood of publication bias may increase if multi-arm studies, perceived as more comprehensive or informative, are more likely to be published, especially if they report more favorable outcomes, potentially influencing the direction of the overall conclusions.

The current review has several strengths. First, the current protocol was registered with PROSPERO. Second, standardized procedures were conducted and the reporting followed the PRISMA guidelines. Third, this is the first network meta-analysis comparing the efficacy of LLLT with different wavelengths for treating KOA symptoms. We utilized reliable methods recommended by the Cochrane Collaboration for the network meta-analysis. Moreover, we employed SUCRA outcomes ratings to identify any subtle differences among these treatments.

This review has several limitations. First, only 13 small-scale studies were included in our analysis. Specifically, the comparison between MWL and L1 was based on one single trial with 65 patients. This limited sample size affected the generalizability of our findings and introduced potential bias. Second, only four included studies evaluated the effects of LLLT on knee physical activity and stiffness. The small sample size and the use of LLLT with wavelength longer than 904–905 nm might lead to non-significant results. Third, the current review only considered the effects of LLLT wavelength on KOA outcomes at the 8-week follow-up. Because other confounders, such as LLLT frequency, or energy density, treatment duration, treatment locations, types of KOA, follow-up time points, may affect the results, our findings should be interpreted with great caution. Future studies should use standardized protocol with different dosages to systematically evaluate the effects of wavelength, frequency, and energy density of LLLT on various clinical outcomes of patients with KOA in short- and long-term follow-ups. Fourth, the SD values from one RCT included in the analysis were derived from images through software, which could potentially lead to inaccuracies. That said, the estimated SD values were calculated using the official methods outlined in the Cochrane Handbook for Systematic Reviews of Interventions, and the image data were extracted using the reliable Engauge Digitizer software [34]. Fifth, four studies included in the network analysis for knee pain had a high risk of bias. After excluding these studies in our sensitivity analysis, 904–905 nm remained to be the optimal wavelength for KOA pain reduction; however, there was no significant difference in pain relief between L1 and sham LLLT. Sixth, we only extracted data at or nearest to eight weeks for meta-analyses. Future studies should compare the treatment effects at different follow-up time points. Seventh, As the network meta-analysis was drawn from indirect comparisons, they should be interpreted with care. Further clinical trials should directly compare the effects of different wavelengths on KOA symptoms. Eighth, studies investigating the combination of LLLT with exercise or other treatments for knee KOA were excluded in the current review, which might limit the generalizability of our findings. Ninth, our meta-analysis might have been affected by publication bias. The Egger test indicated significant publication bias among comparisons, and funnel plots showed asymmetry, suggesting that potential omission of smaller studies with non-significant results from the analysis. Tenth, in this study, the knee pain questionnaires used related to the general condition of the knee rather than to particular areas of the knee joint. Considering that knee pain can originate from multiple tissues [64], different depths of knee pain may respond variably to various wavelengths of LLLT. Future research is also needed to investigate the effects of different wavelengths of LLLT on pain in specific areas of the knee joint that are less easily treated. This would contribute to a more comprehensive understanding of the applications of LLLT in targeting different sources and locations of knee pain. Eleventh, our meta-analysis only included studies published in English, which may introduce language bias and limit the comprehensiveness of our findings.

## Conclusions

Our systematic review and meta-analysis found low certainty of evidence that LLLT could effectively reduce KOA pain, and LLLT with wavelengths 904–905 nm might be the most effective wavelength range for relieving KOA pain. Our findings provided preliminary support for using LLLT to treat KOA pain, but not for improving knee function or stiffness. Future studies should systematically evaluate the influences of different treatment parameters on modifying the clinical outcomes of patients with KOA.

## Supplementary Information

Below is the link to the electronic supplementary material.

Supplementary file1 (PDF 1379 KB)

## Data Availability

The original contributions presented in the study are included in the article/supplementary material. Further inquiries can be directed to the corresponding authors.
