# Supplementary material for: A systematic review and network meta-analysis on the optimal wavelength of low-level light therapy (LLLT) in treating knee osteoarthritis symptoms
Source: Aging Clin Exp Res. 2024 Oct 5;36(1):203. doi: 10.1007/s40520-024-02853-0 (PMC11455796; doi:10.1007/s40520-024-02853-0)
Supplement: Supplementary file 1 — Supplementary file1 (PDF 1379 KB) [file 40520_2024_2853_MOESM1_ESM.pdf]

Supplementary Materials

Title: A systematic review and network meta-analysis on the optimal wavelength of low-level light therapy (LLLT) in treating knee osteoarthritis symptoms

Contents

Supplementary Table 1. Search strategies in four databases.

Supplementary Table 2. GRADE certainty of evidence for LLLT on knee pain.

Supplementary Table 3. Split between direct and indirect evidence of different wavelengths of LLLT for knee pain.

Supplementary Figure 1. Funnel plot representing publication bias for LLLT on knee pain.

Supplementary Figure 2. Forest plot of network meta-analysis of different wavelength of LLLT for knee function.

Supplementary Figure 3. Forest plot of network meta-analysis of different wavelength of LLLT for knee stiffness.

Supplementary Figure 4. Forest plot of direct evidence from direct comparisons for knee pain after excluding high risk studies.

Supplementary Figure 5. A forest plot of network meta-analysis of different wavelengths of LLLT for knee pain after excluding high risk studies.

Supplementary Figure 6. Forest plot of direct evidence from direct comparisons for knee function.

Supplementary Figure 7. Forest plot of direct evidence from direct comparisons for knee stiffness.

Supplementary Figure 8. Forest plot of network meta-analysis of different wavelength of LLLT for knee pain.

Supplementary Table 1. Search strategies in four databases.

**PubMed**		Paper number
#1	"Osteoarthritis"[Mesh]	75,339
#2	OR "Osteoarthritis"[Title/Abstract] OR "Osteoarthritides"[Title/Abstract] OR "Osteoarthrosis"[Title/Abstract] OR "Osteoarthroses"[Title/Abstract] OR "Arthrosis"[Title/Abstract] OR "Arthroses"[Title/Abstract] OR "Degenerative Arthritides"[Title/Abstract] OR "Degenerative Arthritis"[Title/Abstract] OR "Osteoarthrosis Deformans"[Title/Abstract]	92,810
#3	"Low-Level Light Therapy"[Mesh] OR "Lasers, Semiconductor"[Mesh] OR "Laser Therapy"[Mesh] OR "Phototherapy"[Mesh]	110,595
#4	OR "Low-Level Light" [Title/Abstract] OR "Low Level Light" [Title/Abstract] OR "Photobiomodulation"[Title/Abstract] OR "LLLT"[Title/Abstract] OR "Low-Level Laser"[Title/Abstract] OR "Low Level Laser"[Title/Abstract] OR "Low-Power Laser"[Title/Abstract] OR "Low Power Laser"[Title/Abstract] OR "low-intensity laser"[Title/Abstract] OR "low intensity laser"[Title/Abstract] OR "Laser Biostimulation"[Title/Abstract] OR "Phototherapy"[Title/Abstract] OR "Light-Emitting Diode"[Title/Abstract] OR "Light Emitting Diode"[Title/Abstract] OR "laser therapy"[Title/Abstract] OR "high-intensity laser"[Title/Abstract]	32,774

	OR "high intensity laser"[Title/Abstract] OR "high-power laser"[Title/Abstract] OR "high power laser"[Title/Abstract] OR "high-level laser"[Title/Abstract] OR "high level laser"[Title/Abstract]	
#5	"randomized controlled trial"[Publication Type] OR "controlled clinical trial"[Publication Type] OR "randomized"[Title/Abstract] OR "randomised"[Title/Abstract] OR "randomization"[Title/Abstract] OR "randomisation"[Title/Abstract] OR "randomly"[Title/Abstract] OR "placebo"[Title/Abstract] OR "trial"[Title/Abstract]	1,668,089
(#1 OR #2) AND (#3 OR #4) AND #5	(("Osteoarthritis"[Mesh] OR "Osteoarthritis"[Title/Abstract] OR "Osteoarthritides"[Title/Abstract] OR "Osteoarthrosis"[Title/Abstract] OR "Osteoarthroses"[Title/Abstract] OR "Arthrosis"[Title/Abstract] OR "Arthroses"[Title/Abstract] OR "Degenerative Arthritides"[Title/Abstract] OR "Degenerative Arthritis"[Title/Abstract] OR "Osteoarthrosis Deformans"[Title/Abstract]) AND ("Low-Level Light Therapy"[Mesh] OR "Lasers, Semiconductor"[Mesh] OR "Laser Therapy"[Mesh] OR "Phototherapy"[Mesh] OR "Low-Level Light" [Title/Abstract] OR "Low Level Light" [Title/Abstract] OR "Photobiomodulation"[Title/Abstract] OR "LLLT"[Title/Abstract] OR "Low-Level Laser"[Title/Abstract] OR "Low Level Laser"[Title/Abstract] OR "Low-Power Laser"[Title/Abstract] OR "Low Power Laser"[Title/Abstract] OR "low-intensity laser"[Title/Abstract] OR "low intensity laser"[Title/Abstract] OR "Laser Biostimulation"[Title/Abstract] OR "Phototherapy"[Title/Abstract] OR "Light-Emitting Diode"[Title/Abstract] OR "Light Emitting Diode"[Title/Abstract] OR "laser therapy"[Title/Abstract] OR "high-intensity laser"[Title/Abstract] OR "high intensity laser"[Title/Abstract] OR "high-	144

	power laser"[Title/Abstract] OR "high power laser"[Title/Abstract] OR "high-level laser"[Title/Abstract] OR "high level laser"[Title/Abstract])) AND ("randomized controlled trial"[Publication Type] OR "controlled clinical trial"[Publication Type] OR "randomized"[Title/Abstract] OR "randomised"[Title/Abstract] OR "randomization"[Title/Abstract] OR "randomisation"[Title/Abstract] OR "randomly"[Title/Abstract] OR "placebo"[Title/Abstract] OR "trial"[Title/Abstract])	
**Embase**		
#1	'low level laser therapy'/exp OR 'phototherapy'/exp	110,429
#2	'light therapy':ab,ti OR 'low level light':ab,ti OR 'photobiomodulation':ab,ti OR 'lllt':ab,ti OR 'low-level laser':ab,ti OR 'low level laser therapy':ab,ti OR 'low-power laser':ab,ti OR 'low power laser':ab,ti OR 'low-intensity laser':ab,ti OR 'low intensity laser':ab,ti OR 'laser biostimulation':ab,ti OR 'laser phototherapy':ab,ti OR 'Light-Emitting Diode':ab,ti OR 'Light Emitting Diode':ab,ti OR 'laser therapy':ab,ti OR 'high-intensity laser':ab,ti OR 'high intensity laser':ab,ti OR 'high-power laser':ab,ti OR 'high power laser':ab,ti OR 'high-level laser':ab,ti OR 'high level laser':ab,ti	27,476
#3	'osteoarthritis'/exp	158,498
#4	('osteoarthritis' OR osteoarthritis:ti,ab,kw OR osteoarthritides:ti,ab,kw OR osteoarthrosis:ti,ab,kw OR osteoarthroses:ti,ab,kw OR arthrosis:ti,ab,kw OR arthroses:ti,ab,kw) AND [english]/lim	116,579
#5	'randomized controlled trial'/exp OR 'randomized controlled trial':ti,ab,it OR 'randomized':ti,ab,it OR 'randomised':ti,ab,it OR 'randomization':ti,ab,it OR 'randomisation':ti,ab,it OR 'rct':ti,ab,it OR 'randomly':ti,ab,it OR	1,878,522

	'placebo':ti,ab,it	
(#1 OR #2) AND (#3 OR #4) AND #5		238
**Web of Science**		
#1	(((TS=(osteoarthritis)) OR TI=("osteoarthritis" OR "Osteoarthritides" OR "Osteoarthrosis" OR "Osteoarthroses" OR "Arthrosis" OR "Arthroses" OR "Degenerative Arthritides" OR "Degenerative Arthritis" OR "Osteoarthrosis Deformans")) OR AB=("osteoarthritis" OR "Osteoarthritides" OR "Osteoarthrosis" OR "Osteoarthroses" OR "Arthrosis" OR "Arthroses" OR "Degenerative Arthritides" OR "Degenerative Arthritis" OR "Osteoarthrosis Deformans")) OR AK=("osteoarthritis" OR "Osteoarthritides" OR "Osteoarthrosis" OR "Osteoarthroses" OR "Arthrosis" OR "Arthroses" OR "Degenerative Arthritides" OR "Degenerative Arthritis" OR "Osteoarthrosis Deformans")	117,350
#2	(((TS=("Low-Level Light Therapy" OR "Lasers, Semiconductor" OR "Laser Therapy" OR "Phototherapy" OR "Light-Emitting Diode" OR "Light Emitting Diode")) OR TI=("Low-Level Light" OR "Low Level Light" OR "Photobiomodulation" OR "LLLT" OR "Low-Level Laser" OR "Low Level Laser" OR "Low-Power Laser" OR "Low Power Laser" OR "Low-intensity Laser" OR "Low intensity Laser" OR "Laser Biostimulation" OR "Laser	1,024,838

	Phototherapy" OR "Laser" OR "Light-Emitting Diode" OR "Light Emitting Diode" OR "High-Level Light" OR " High Level Light" OR " High-Level Laser" OR "High Level Laser" OR "High-Power Laser" OR "High Power Laser" OR "High-intensity Laser" OR "High intensity Laser")) OR AB=("Low-Level Light" OR "Low Level Light" OR "Photobiomodulation" OR "LLLT" OR "Low-Level Laser" OR "Low Level Laser" OR "Low-Power Laser" OR "Low Power Laser" OR "Low-intensity Laser" OR "Low intensity Laser" OR "Laser Biostimulation" OR "Laser Phototherapy" OR "Laser" OR "Light-Emitting Diode" OR "Light Emitting Diode" OR "High-Level Light" OR " High Level Light" OR " High-Level Laser" OR "High Level Laser" OR "High-Power Laser" OR "High Power Laser" OR "High-intensity Laser" OR "High intensity Laser")) OR AK=("Low-Level Light" OR "Low Level Light" OR "Photobiomodulation" OR "LLLT" OR "Low-Level Laser" OR "Low Level Laser" OR "Low-Power Laser" OR "Low Power Laser" OR "Low-intensity Laser" OR "Low intensity Laser" OR "Laser Biostimulation" OR "Laser Phototherapy" OR "Laser" OR "Light-Emitting Diode" OR "Light Emitting Diode" OR "High-Level Light" OR " High Level Light" OR " High-Level Laser" OR "High Level Laser" OR "High-Power Laser" OR "High Power Laser" OR "High-intensity Laser" OR "High intensity Laser")	
#3	TS=(Randomized controlled trial)	498,279
#1 AND #2 #3		119
Cochrane Library		

#1	MeSH descriptor: [Osteoarthritis] explode all trees	9570
#2	("Osteoarthritis" OR "Osteoarthritides" OR "Osteoarthrosis" OR "Osteoarthroses" OR "Arthrosis" OR "Arthroses" OR "Degenerative Arthritides" OR "Degenerative Arthritis" OR "Osteoarthrosis Deformans"):ti,ab,kw	22,113
#3	MeSH descriptor: [Low-Level Light Therapy] explode all trees OR<br>MeSH descriptor: [Lasers, Semiconductor] explode all trees OR<br>MeSH descriptor: [Laser Therapy] explode all trees OR MeSH descriptor: [Phototherapy] explode all trees	7,927
#4	("Low-Level Light" OR "Low Level Light" OR "Photobiomodulation" OR "LLLT" OR "Low-Level Laser" OR "Low Level Laser" OR "Low-Power Laser" OR "Low Power Laser" OR "Low-intensity Laser" OR "Low intensity Laser" OR "Laser Biostimulation" OR "Phototherapy" OR "Laser Phototherapy" OR "Laser" OR "Light-Emitting Diode" OR "Light Emitting Diode" OR "High-Level Laser" OR "High Level Laser" OR "High-Power Laser" OR "High Power Laser" OR "High-intensity Laser" OR "High intensity Laser"):ti,ab,kw	26,087
	(#1 OR #2) AND (#3 OR #4)	273

Supplementary Table 2 GRADE certainty of evidence for LLLT on knee pain.

Certainty assessment							№ of patients		Effect		Certainty
**№ of studies**	**Study design**	**Risk of bias**	**Inconsistency**	**Indirectness**	**Imprecision**	**Other considerations**	**LLLT**	**sham LLLT**	**Relative (95% CI)**	**Absolute (95% CI)**	
Conventional meta-analysis (Overall LLLT compare with sham LLLT)											
13	randomised trials	serious[a]	serious[b]	not serious[c]	not serious	publication bias strongly suspected strong association[c]	342	300	SMD **0.96 SD higher** (0.31 higher to 1.61 higher)		⊕⊕◯◯ Low
NMA estimate on MWL vs. Sham											
4	randomised trials	not serious[a]	not serious	very serious[d]	serious[e]	none	125	120	SMD **0.83 SD higher** (-0.06 lower to 1.72 higher)		⊕◯◯◯ Very low
NMA estimate on L2 vs. Sham											
3	randomised trials	not serious	serious[f]	very serious[d]	not serious	strong association	84	81	SMD **1.42 SD higher** (0.31 higher to 2.53 higher)		⊕⊕◯◯ Low
NMA estimate on L1 vs. Sham											
7	randomised trials	serious[a]	serious[g]	very serious[d]	not serious	strong association	133	130	SMD **0.81 SD higher** (0.11 higher to 1.50 higher)		⊕◯◯◯ Very low

CI: confidence interval; SMD: standardised mean difference; LLLT: low-level light therapy; L1: 785-850 nm LLLT; L2: 904-905 nm LLLT; MWL: multi-wavelength LLLT; Sham: sham LLLT; NMA: network meta-analysis; GRADE: grading of recommendations, assessment, development, and evaluation

Explanations: a. One study is high risk due to missing outcome data; b. I^2 is 86%; c. P-value for Egger regression test of funnel plot asymmetry is 0.0376; d. From network meta-analysis
e. 95% CI includes 0; f. I^2 is 95%; g. I^2 is 88.8%

Supplementary Table 3. Split between direct and indirect evidence of different wavelengths of LLLT for knee pain.

**Arm 1**	**Arm 2**	**k**	**I^2**	**Direct estimate#**	**Indirect estimate#**	**Network meta-analysis#**	**Incoherence**
L1	L2	0	.	.	-0.61 [-1.92; 0.70]	-0.61 [-1.92; 0.70]	.
L1	MWL	1	.	-0.64 [-2.46; 1.17]	0.29 [-1.01; 1.60]	-0.02 [-1.08; 1.04]	0.41
L1	Sham	7	88.8%	**0.84 [0.13; 1.55]**	-0.25 [-4.03; 3.54]	**0.81 [0.11; 1.50]**	0.58
L2	MWL	0	.	.	0.59 [-0.83; 2.01]	0.59 [-0.83; 2.01]	.
L2	Sham	3	95.0%	**1.42 [0.31; 2.53]**	.	1.42 [0.31; 2.53]	.
MWL	Sham	4	34.2%	0.74 [-0.18; 1.65]	2.24 [-1.27; 5.75]	**0.83 [-0.06; 1.72]**	0.42

#Data are shown as standardized mean difference (SMD) and 95% credible intervals.

LLLT: low-level light therapy; L1: 785-850 nm LLLT; L2: 904-905 nm LLLT; MWL: multi-wavelength LLLT; Sham: sham LLLT;

Bolds are statistically significant.

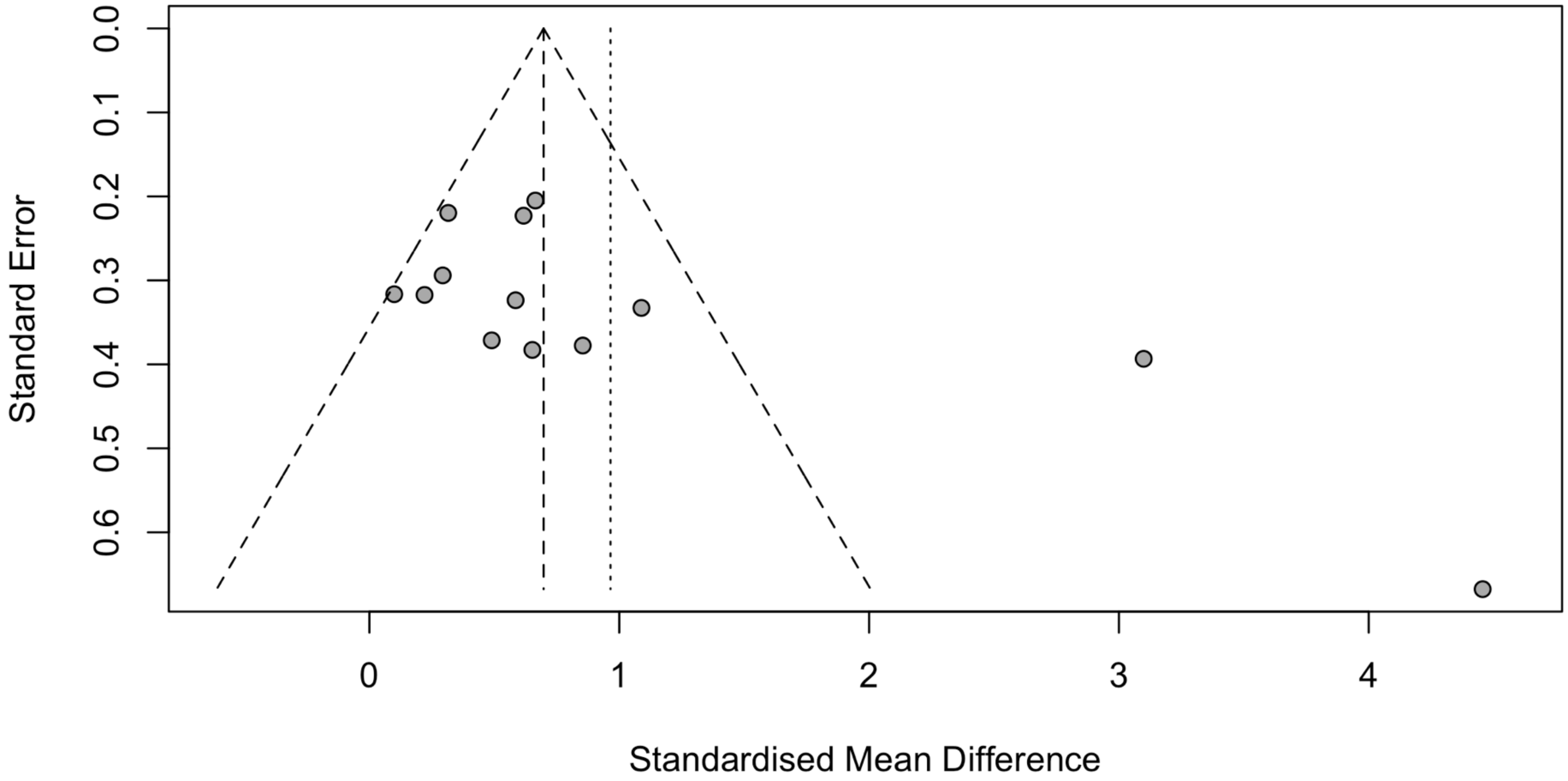

Supplementary Figure 1. Funnel plot representing publication bias for low-level light therapy on knee pain.

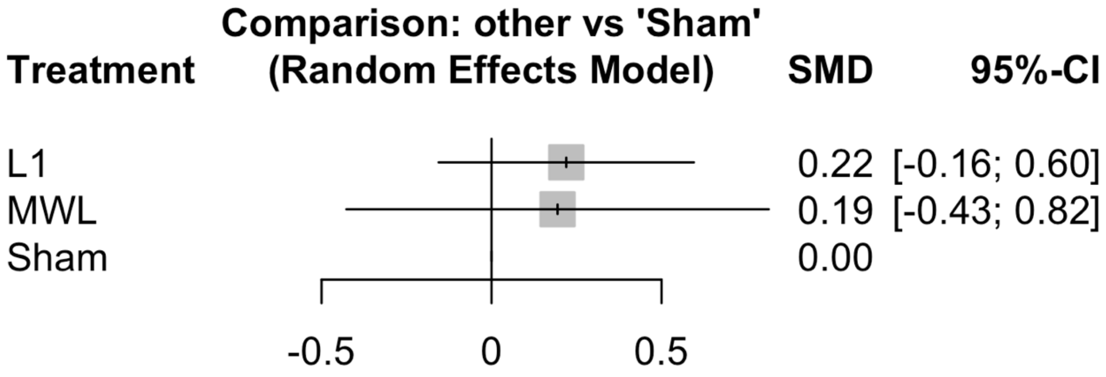

Supplementary Figure 2. Forest plot of network meta-analysis of different wavelength of LLLT for knee function.

LLLT: low-level light therapy; L1: 785-850 nm LLLT; MWL: multi-wavelength LLLT; Sham: sham LLLT; SMD: standardized mean difference

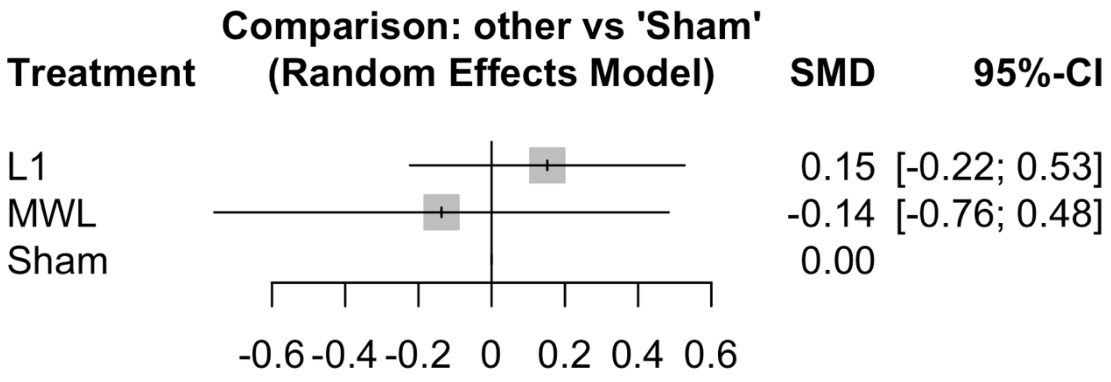

Supplementary Figure 3. Forest plot of network meta-analysis of different wavelength of LLLT for knee stiffness.

LLLT: low-level light therapy; L1: 785-850 nm LLLT; MWL: multi-wavelength LLLT; Sham: sham LLLT; SMD: standardized mean difference

Study	Experimental Total	Experimental Mean	Experimental SD	Control Total	Control Mean	Control SD	SMD	95%-CI	Weight (common)	Weight (random)
Ahmad Alghadir 2014	20	5.90	3.0281	20	4.10	3.0023	0.59	[-0.05; 1.22]	11.1%	11.3%
Dwi R. Helianthi 2016	30	40.50	14.8000	29	1.30	6.0000	3.40	[2.59; 4.22]	6.7%	11.0%
Funda Tascioglu 2004	20	0.40	3.7474	20	0.29	4.1703	0.03	[-0.59; 0.65]	11.6%	11.3%
Gopal mbi S 2017	17	6.60	1.1136	17	0.80	1.4107	4.46	[3.15; 5.76]	2.6%	9.8%
Patricia Gabrielli Vass 2020	14	2.59	4.5110	15	1.15	4.0793	0.33	[-0.41; 1.06]	8.3%	11.1%
Punpetch Sirirat 2022	21	4.14	1.4271	21	2.21	2.0033	1.09	[0.44; 1.74]	10.5%	11.3%
Reta Alqualo-Costa 2021	42	2.10	3.0610	42	1.10	3.2187	0.32	[-0.12; 0.75]	24.1%	11.6%
Vanessa Ovanessian Fukuda 2011	25	1.70	2.7622	22	0.90	2.5865	0.29	[-0.28; 0.87]	13.5%	11.4%
Xueyong Shen 2009	20	4.18	3.2832	20	3.47	3.0188	0.22	[-0.40; 0.84]	11.6%	11.3%
**Common effect model**	**209**			**206**			**0.70**	**[0.48; 0.91]**	**100.0%**	**--**
**Random effects model**							**1.13**	**[0.15; 2.12]**	**--**	**100.0%**

Heterogeneity: $I^2 = 91\%$, $\tau^2 = 2.1293$, $p < 0.01$

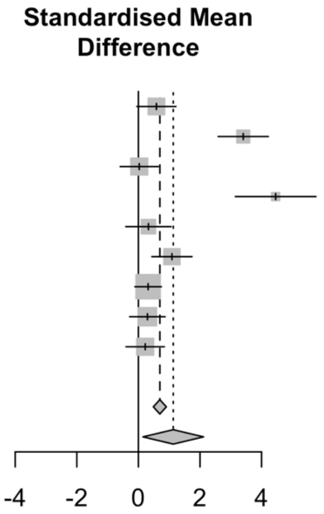

Supplementary Figure 4. Forest plot of direct evidence from direct comparisons for LLLT on knee pain after excluding high risk studies.

LLLT: low-level light therapy; SMD: standardized mean difference; SD: standard division

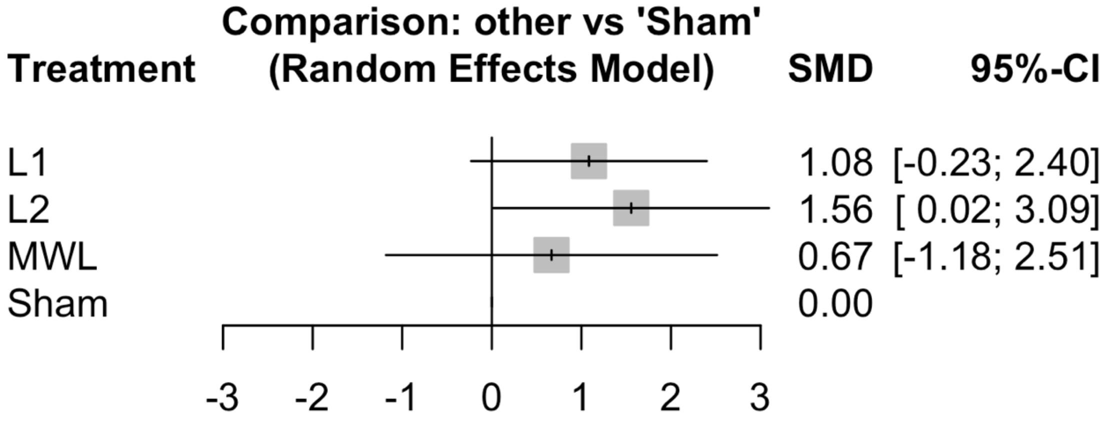

Supplementary Figure 5. A forest plot of network meta-analysis of different wavelengths of LLLT for knee pain after excluding high risk studies. LLLT: low-level light therapy; L1: 785-850 nm LLLT; L2: 904-905 nm LLLT; MWL: multi-wavelength LLLT; Sham: sham LLLT; SMD: standardized mean difference

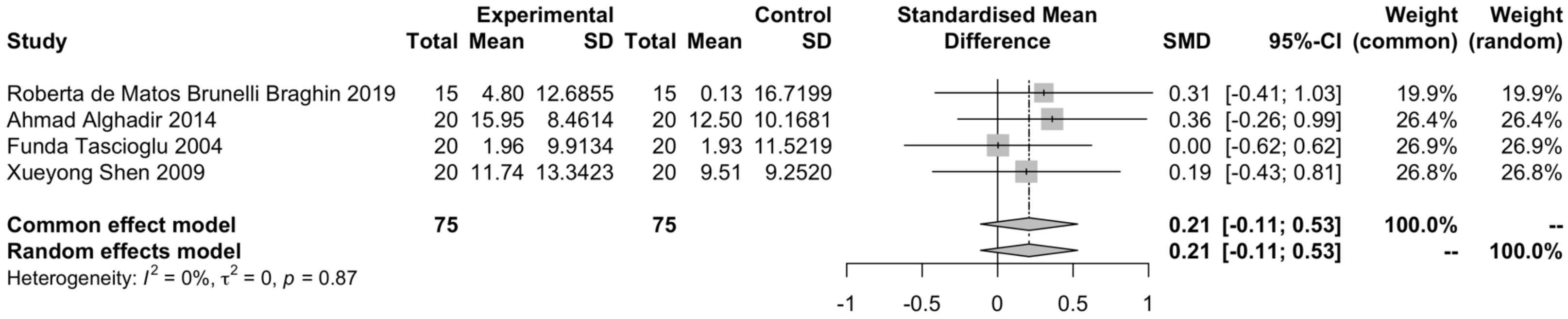

Supplementary Figure 6. Forest plot of direct evidence from direct comparisons for knee function.

SMD: standardized mean difference; SD: standard division

Study	Experimental Total	Experimental Mean	Experimental SD	Control Total	Control Mean	Control SD	SMD	95%-CI	Weight (common)	Weight (random)
Roberta de Matos Brunelli Braghin 2019	15	0.53	1.9391	15	-0.40	2.2076	0.44	[-0.29; 1.16]	19.6%	19.6%
Ahmad Alghadir 2014	20	1.85	1.5639	20	1.55	2.1301	0.16	[-0.46; 0.78]	26.8%	26.8%
Funda Tascioglu 2004	20	-0.08	1.7984	20	0.07	2.2339	-0.07	[-0.69; 0.55]	26.8%	26.8%
Xueyong Shen 2009	20	0.68	1.7485	20	0.90	1.4486	-0.13	[-0.75; 0.49]	26.8%	26.8%
**Common effect model**	**75**			**75**			**0.07**	**[-0.25; 0.39]**	**100.0%**	**--**
**Random effects model**							**0.07**	**[-0.25; 0.39]**	**--**	**100.0%**

Heterogeneity: $I^2 = 0\%$, $\tau^2 = 0$, $p = 0.64$

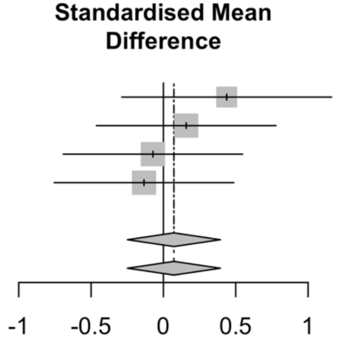

Supplementary Figure 7. Forest plot of direct evidence from direct comparisons for LLLT on knee stiffness.

LLLT: low-level light therapy; SMD: standardized mean difference; SD: standard division

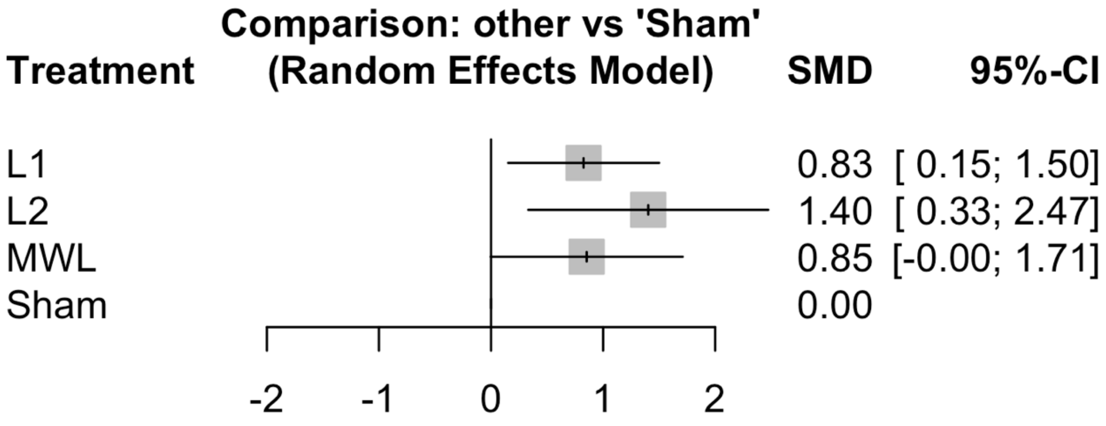

Supplementary Figure 8. Forest plot of network meta-analysis of different wavelength of LLLT for knee pain.

LLLT: low-level light therapy; L1: 785-850 nm LLLT; L2: 904-905 nm LLLT; MWL: multi-wavelength LLLT; Sham: sham LLLT; SMD: standardized mean difference
